# High-throughput micro-nanostructuring by microdroplet inkjet printing

**DOI:** 10.3762/bjnano.9.222

**Published:** 2018-09-04

**Authors:** Hendrikje R Neumann, Christine Selhuber-Unkel

**Affiliations:** 1 Biocompatible Nanomaterials, Institute for Materials Science, University of Kiel, Kaiserstr. 2, 24143 Kiel, Germany

**Keywords:** biofunctional surfaces, inkjet printing, microstructures, nanolithography, nanoparticles

## Abstract

The production of micrometer-sized structures comprised of nanoparticles in defined patterns and densities is highly important in many fields, ranging from nano-optics to biosensor technologies and biomaterials. A well-established method to fabricate quasi-hexagonal patterns of metal nanoparticles is block copolymer micelle nanolithography, which relies on the self-assembly of metal-loaded micelles on surfaces by a dip-coating or spin-coating process. Using this method, the spacing of the nanoparticles is controlled by the size of the micelles and by the coating conditions. Whereas block copolymer micelle nanolithography is a high-throughput method for generating well-ordered nanoparticle patterns at the nanoscale, so far it has been inefficient in generating a hierarchical overlay structure at the micrometer scale. Here, we show that by combining block copolymer micelle nanolithography with inkjet printing, hierarchical patterns of gold nanoparticles in the form of microstructures can be achieved in a high-throughput process. Inkjet printing was used to generate droplets of the micelle solution on surfaces, resulting in printed circles that contain patterns of gold nanoparticles with an interparticle spacing between 25 and 42 nm. We tested this method on different silicon and nickel–titanium surfaces and the generated patterns were found to depend on the material type and surface topography. Based on the presented strategy, we were able to achieve patterning times of a few seconds and produce quasi-hexagonal micro-nanopatterns of gold nanoparticles on smooth surfaces. Hence, this method is a high-throughput method that can be used to coat surfaces with nanoparticles in a user-defined pattern at the micrometer scale. As the nanoparticles provide a chemical contrast on the surface, they can be further functionalized and are therefore highly relevant for biological applications.

## Introduction

Many applications require well-organized micro- and nanoscale patterning of metallic nanoparticles. Examples include high-performance optics [1], multimodal waveguides [2], biosensors [3] and biomaterials [4]. Using electron-beam lithography, it is possible to generate such patterns with very high spatial precision [5]. Focused electron beam induced deposition (FEBID) even serves as a method to deposit 3D nanostructures without the need of masks [6]. A further and very successful method to write gold nanoparticle structures is dip-pen nanolithography [7]. Although all these methods are highly precise, it would be expensive and time consuming to use them to coat large areas on the centimeter scale.

A convenient, high-throughput method to achieve hexagonal patterns of metal nanoparticles with a well-defined spacing between a few tens to several hundreds of nanometers is block copolymer micelle nanolithography (BCML) [8]. This technique is based on the self-assembly of metal-containing micelles on surfaces during dip-coating or spin-coating. BCML is very efficient in coating large areas with nanoparticles in quasi-hexagonal arrays. The spacing between the nanoparticles is controlled by the block copolymer used for forming the micelles and by the coating conditions, e.g., spin-coating and dip-coating parameters. BCML has been realized for preparing arrays of different types of nanoparticles, including gold [9], titania [10], and hybrid nanoparticles [11–12]. If the nanoparticles are intended to provide a chemical contrast for further functionalization, gold nanoparticles are an appropriate choice as they are easily functionalized using thiol chemistry [13]. For example, gold nanoparticles prepared by BCML can be biofunctionalized such that they serve as biomimetic anchorage sites for cell adhesion molecules, whereby their spacing has been shown to be highly decisive for cell adhesion [14]. Still, the anchorage site spacing required for cell adhesion depends on the chemistry of a particular adhesion ligand [15]. It has even been reported that cells can respond to differences in ligand spacing as small as 1 nm across the cell diameter [16]. Therefore, the fabrication of complex patterns of gold nanoparticles is highly relevant in the context of the biomimicry of cell adhesion environments.

Whereas BCML can intrinsically only coat surfaces with nanoparticle arrays, many biosensor applications also require well-defined structures and concentrations of ligands in microarrays [17]. Particularly the generation of cell arrays is a highly challenging task [18–19]. A feasible strategy would therefore be to combine the benefits of BCML, i.e., the highly controllable generation of regular nanoparticle patterns, with methods that generate an overlay microstructure. In recent years, several methods have been proposed to fabricate such structures, but all of them require several complicated process steps that are only achievable with clean-room methods. For example, so-called “micro-nanostructures” have been fabricated by combining BCML with electron-beam lithography and photolithography [20–21]. A different approach was proposed based on topography-induced micro-nanostructuring, but this method requires a nanotopographically structured substrate and can only provide gold nanoparticles of different spacing in areas next to each other, not in distinct free areas [22]. Hence, none of the methods reported so far is a single-step method that can be carried out in a standard lab without the need for clean-room equipment.

In contrast to such methods, inkjet printing has recently become a powerful and affordable tool for the quick, easy-to-handle and user-defined surface patterning in various orders of magnitude and with a broad spectrum of different inks, including conductive gels, dispersions, but also proteins [23–27]. In this way, even flexible materials can easily be patterned [28]. Therefore, with this method, we have the combined benefits of BCML with the advantages of inkjet printing to achieve nanoparticle structures in defined microarrays. Such micro-nanostructures have been generated on different types of silicon and nickel–titanium (NiTi) materials, thus providing a novel method to micro-nanostructure and functionalize materials, which are relevant in biomaterial and biosensor applications.

## Results and Discussion

### Inkjet printing for generating micelle solution droplets

**Figure 1:**
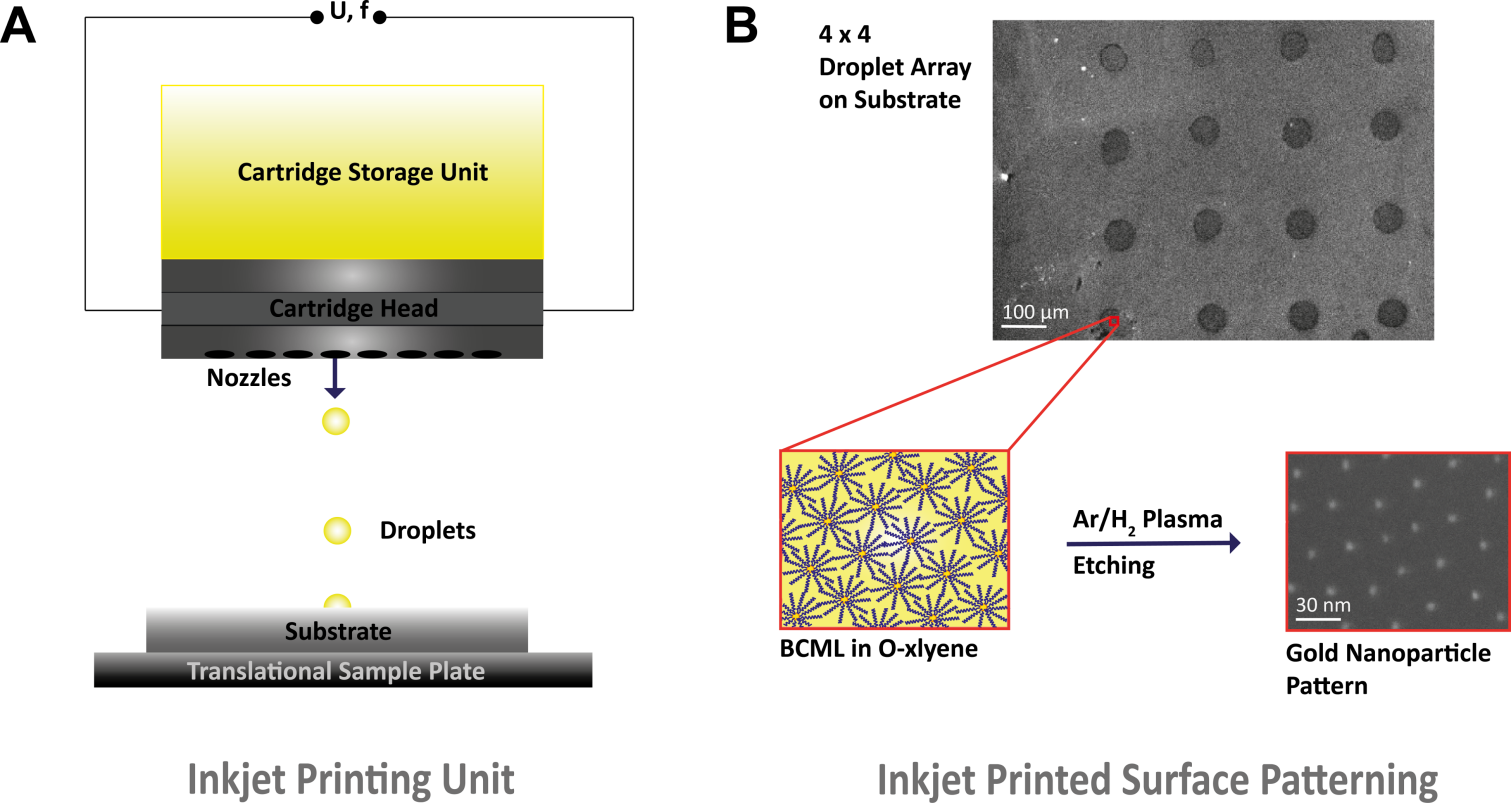
(A) Schematic representation of the operational principle of the Fuji Dimatix DMP-2800 printing head. 4 mL of gold-loaded micelle solution (BMCL) is stored in the cartridge storage unit that is connected to the cartridge head. Via the piezoelectrically driven nozzles, droplets are generated whose shape is controlled via the voltage and frequency of each nozzle. In this way, droplets are generated on the different substrates. (B) User-defined microdroplet patterns are generated on the substrate via the movable printing head. The droplets of gold-containing micelle solution (BCML in *o*-xylene) are plasma-etched and the decoration of the surface with gold nanoparticles is then finished.

**Figure 2:**
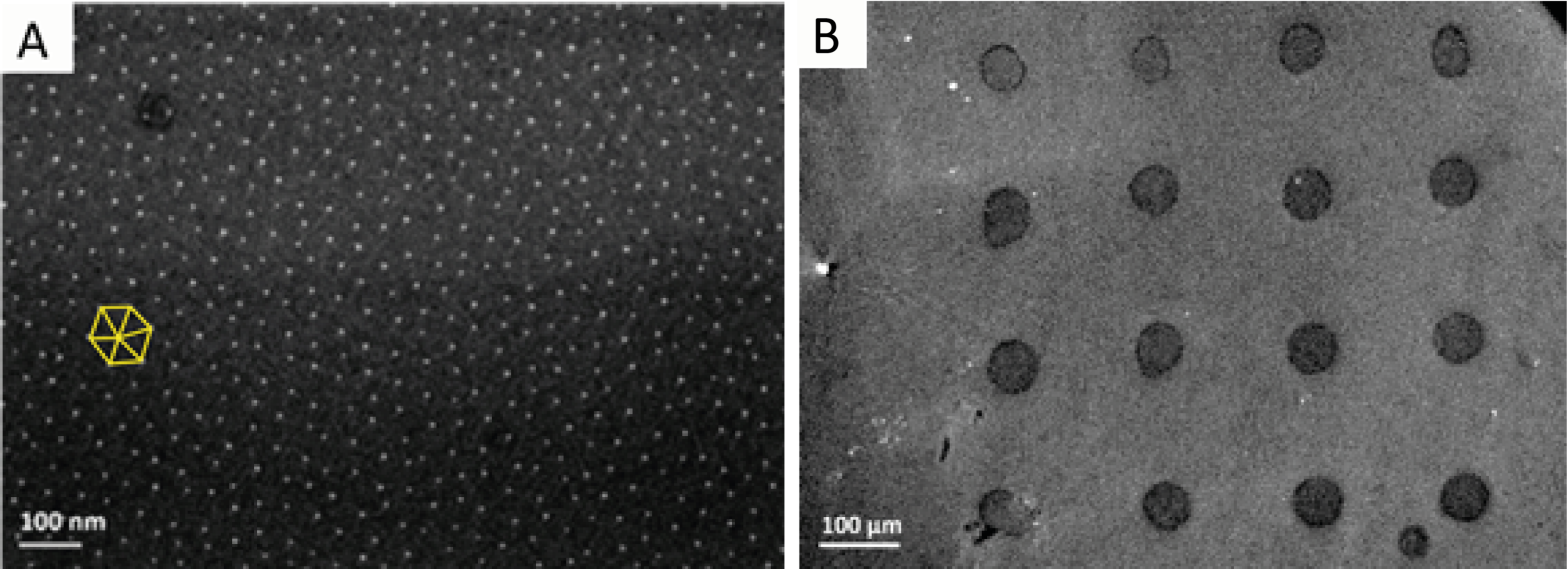
(A) Pattern of gold nanoparticles on the reference sample after spin coating of a 10 × 10 mm silicon wafer unit with a gold-containing micelle solution at 7000 rpm and Ar/H_2_ plasma etching at 300 W for 1 h. An exemplary quasi-hexagon pattern is drawn (yellow) for visualization. (B) Inkjet-printed 4 × 4 droplet pattern after drying and Ar/H_2_ plasma treatment at 300 W for 1 h. Both images were recorded with SEM.

The micellar gold nanoparticle solution was first spin-coated on a poly-silicon (poly-Si) 10 × 10 mm wafer unit to test the properties of the solution and to generate a control sample. For the used block copolymer and concentrations, it is well-known that spherical micelles form [8], hence a closed-packed assembly of the micelles gives rise to hexagonal micelle patterns. As the self-assembly does not lead to a perfect hexagonal ordering of the micelles, the patterns generated with BCML are often referred to as “quasi-hexagonal” patterns [9]. In our experiments, quasi-hexagonal patterns of gold nanoparticles were achieved on poly-Si with a mean interparticle distance of 32.6 ± 3.2 nm. The solution was then used for inkjet printing 4 × 4 droplet patterns on five different biocompatible substrates, as shown in Figure 1 and Figure 2B for poly-Si. As the cartridge consists of 16 nozzles with an orifice size of 21.5 µm each, and the distance between the nozzles is 254 µm, 16 droplets in a row can be printed at once in less than 1 s. For printing the 4 × 4 matrix shown in Figure 2, a single nozzle was used at a nozzle frequency of about 80 kHz. Including the printer head movement, approximately 16 s were necessary for printing the total 4 × 4 matrix.

Although a plasma process is needed for removing the polymer shell of the micelles, this is not a limitation in our method, as the plasma process is typically carried out for up to 20–30 samples at the same time. The difference in our method compared to other BCML micro-nanostructuring strategies is the time for the patterning itself – it is much shorter and less complicated than other approaches that employ additional steps such as electron-beam lithography or photolithography [20–21]. A further result of the plasma treatment could be a rough surface, which has been reported both for Si, SiO_2_ and NiTi [29–30]. However, as the plasma treatment takes place after the inkjet printing process, a change in roughness will not influence the droplet size, but might influence further functionalization steps.

**Figure 3:**
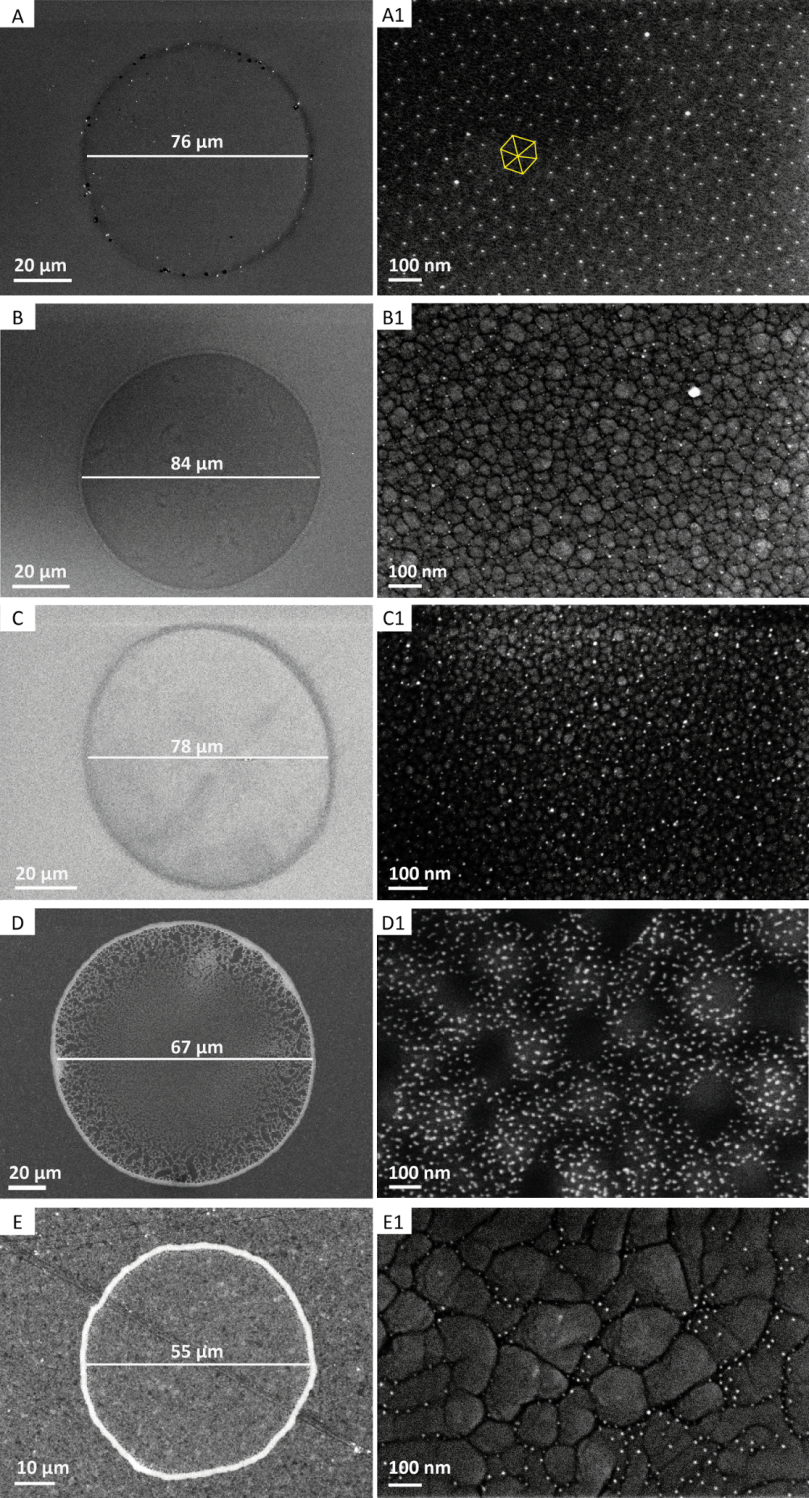
SEM images of size and droplet shape on the five different materials (A–E) and nanodot distribution after Ar/H_2_ plasma etching in the center of each droplet (A1−E1). The substrates are: (A) poly-silicon, (B) amorphous silicon of 200 nm thickness on poly-silicon, (C) amorphous silicon of 400 nm thickness on poly-silicon, (D) 50 µm thick free-standing nickel–titanium foil, and (E) 200 nm nickel–titanium thin film on 4 µm copper and a glass substrate.

The droplet boundary description was set in the machine to 10 µm, but varies depending on the substrate material (see Figure 2 and Figure 3A–E), based on the interaction between fluid and substrate. As a consequence, poly-Si and both of the amorphous silicon (a-Si 200 and a-Si 400) samples have a droplet diameter size in the range of 76 to 84 µm, while the droplet diameter on freestanding NiTi foil and the 200 nm thick sputtered NiTi thin film varies between 55 and 65 µm. These were the smallest possible diameters that could be generated with our setup. The maximum size of the droplets depends on the maximum droplet volume, which can be up to 10 pL per pulse. The silicon nozzle orifice diameter is approximately 21.5 µm, which we did not change in our experiments. It is in principle also possible to write lines and other patterns instead of droplets. In addition, we also assume that the complex rheology of the micelle solution influences the diameter and the boundary of the droplets [31]. It is also clearly visible from Figure 3 that the particle density is not completely homogeneous within a printed circle. On the one hand, this is caused by the surface, on the other hand, we also observe coffee-ring structures [32].

Clearly, the droplet size is different for different materials. The nanoparticle distribution inside the droplet is the more symmetric for samples with smaller grains. For poly-silicon, the gold nanoparticles are arranged in a distorted quasi-hexagonal pattern, while for sample B, C and D, the nanoparticles are mainly situated on the grain boundaries.

### Droplet size is a function of surface roughness

**Figure 4:**
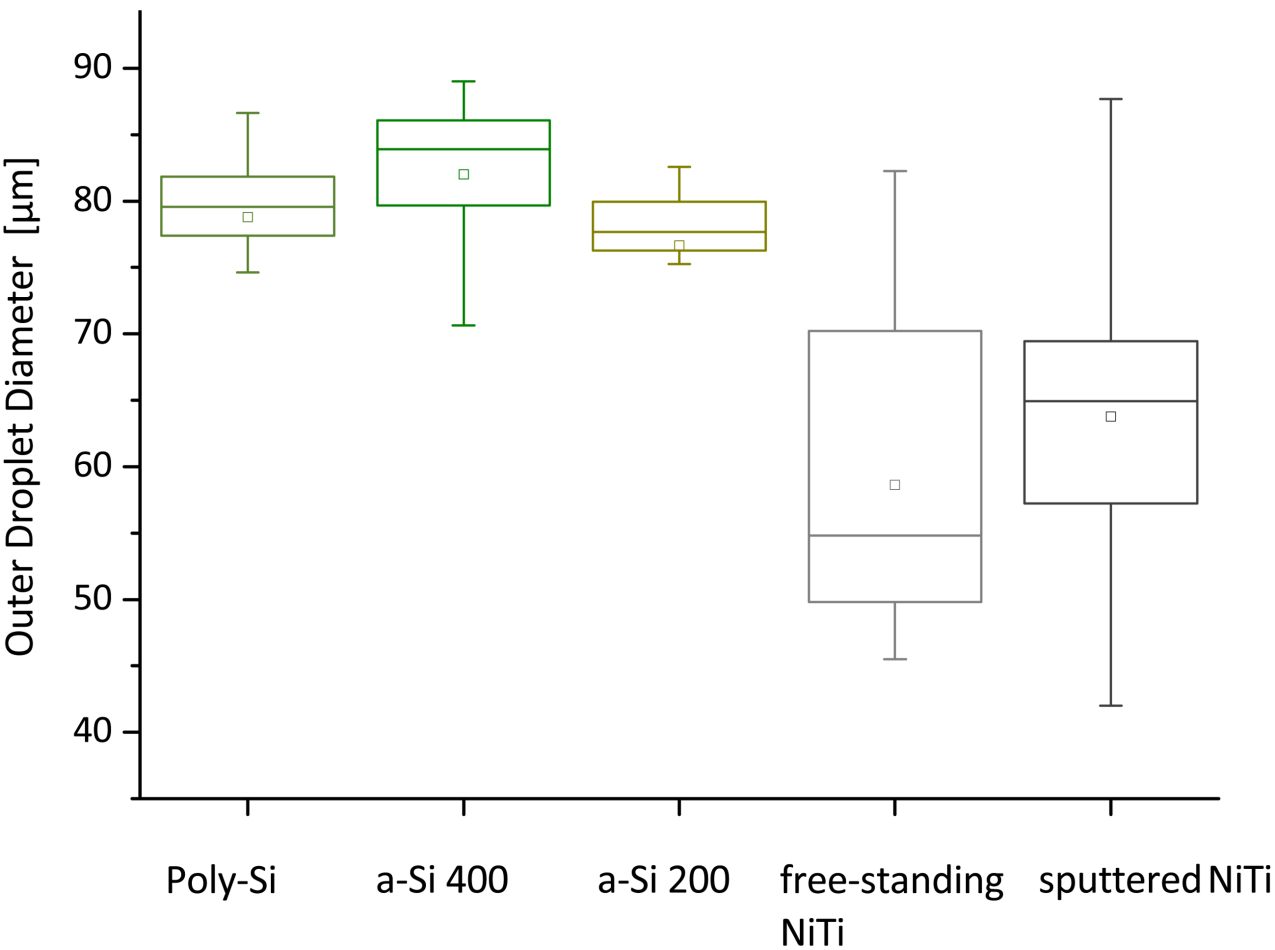
Distribution of the droplet diameter of the micellar gold nanoparticle solution on different materials presented in boxcharts (box: interquartile range; line in each box: median; dot: mean; whiskers: minimum/maximum). The droplet diameter for the poly-silicon (poly-Si), the amorphous silicon of 400 nm thickness (a-Si 400) and the amorphous silicon of 200 nm thickness (a-Si 200) have a comparable mean diameter of around 80 µm. On the other hand, the droplet diameters on a free-standing nickel–titanium (NiTi) foil and a 200 nm thick nickel–titanium thin film sputtered on a 4 µm thick copper layer (sputtered NiTi) have a broad distribution with a mean value of around 60 µm.

Figure 4 presents the diameter distribution of micelle solution droplets for each sample. The droplets on poly-Si have a comparable size with a tight variation range of only about 5 µm. The distribution is symmetric, whereby the mean value of the diameter is nearly the same as the median. Amorphous silicon results in a less symmetric diameter distribution: while 50% of the droplet diameters are within the upper and lower quartiles on a-Si 400 sample and vary around 83 µm, droplet diameters on a-Si 200 sample are slightly shifted to lower mean values of around 77 µm. The medians do not match the mean values in these cases, so the distribution is asymmetric. In general, the micellar solution appears to have a comparable spreading behavior on all silicon samples. In contrast, there is a broad distribution of droplet diameters on the 50 µm thick free-standing NiTi sample: about 50% of the droplets have a diameter between 49 to 70 µm. For the sputtered NiTi thin film, the droplet diameters have a narrower distribution around the mean value of 63 µm, but the distribution has long tails, as shown by the upper whisker maximum and minimum. Apparently, the micelle-containing *o*-xylene solution spreads out most reproducibly on silicon and less on NiTi.

**Figure 5:**
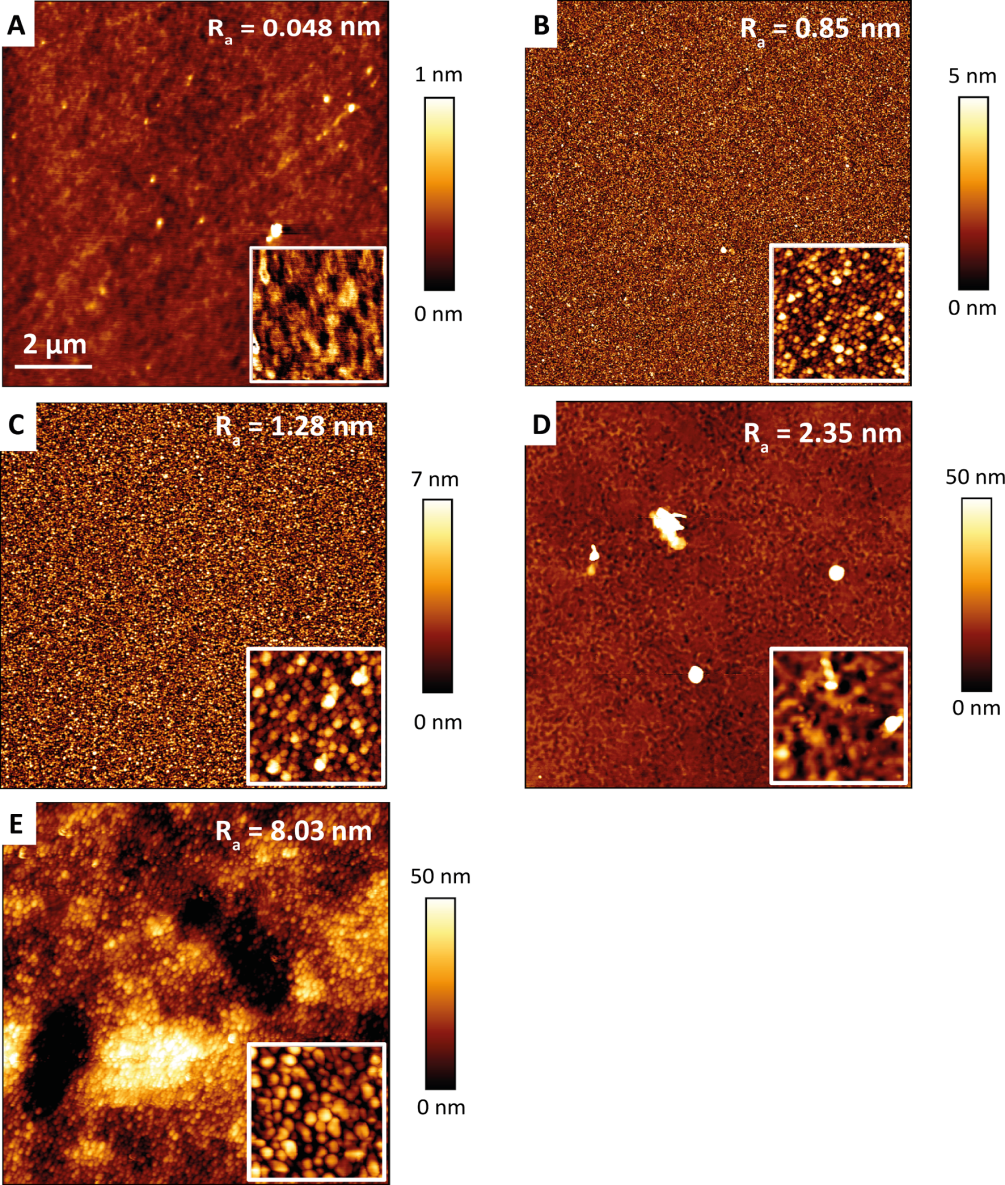
Surface topography imaged by atomic force microscopy for: (A) poly-silicon, (B) amorphous silicon of 400 nm thickness, (C) amorphous silicon of 200 nm thickness, (D) 50 µm thick free-standing NiTi foil in a 10 µm × 10 µm area, and (E) 200 nm NiTi thin film on 4 µm Cu and a glass substrate. The insets were imaged in 1 µm × 1 µm areas. The height distribution is given by the temperature scale bar for each sample. Based on the automatically determined average surface roughness, *R*_a_, for each area, sputtered NiTi (sample shown in (E)) has the highest roughness and poly-silicon (A) is the smoothest sample.

To explain the differences in the droplet diameters of our different materials, the surface topography of the samples was measured using atomic force microscopy (AFM). As shown in Figure 5A, poly-Si has the lowest roughness of 48 pm, followed by a-Si 400 (850 pm), and a-Si 200 (1.25 nm). The NiTi samples have a much higher roughness (freestanding NiTi foil: 2.35 nm), i.e., 6.5 times the roughness of the a-Si 200 sample. Obviously, rougher surfaces lead to smaller droplets. The broad distribution of droplet diameter on the NiTi foil might also be due to dirt particles on the surface as shown by the bright spots in the AFM image in Figure 5D. Another factor that might also influence the droplet diameter is the surface texture of NiTi on copper (Figure 3E).

There is a variety of different techniques known to reduce or eliminate this effect, such as the distinct choice of solvent mixture and concentration [33] or adding nanofibers to colloidal dispersions [34]. In our BCML solution, such adaptations were not possible, partly because of the stabilization of the micellar system by *o*-xylene at a fixed concentration.

### Gold nanoparticle distribution depends on the material

**Figure 6:**
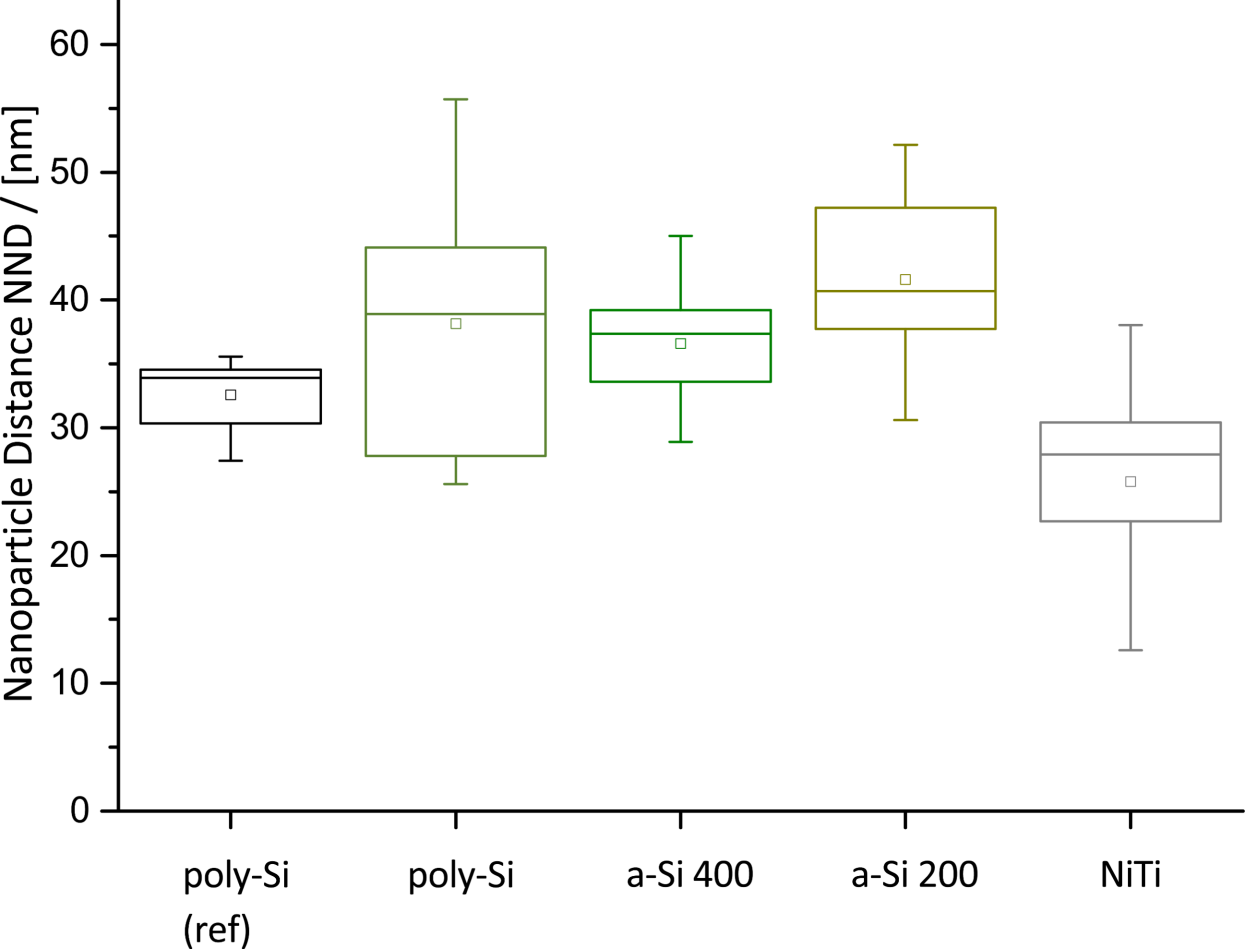
Boxchart of nanoparticle separation distance in the center of microcircles for each substrate material, as determined by a nearest neighbor distance (NND) algorithm. Each measurement is based on up to 40 images analyzed for a total of 60,000 nanoparticles. The nanoparticle separation distance on spin-coated poly-silicon is added as a reference sample and has the smallest standard deviation, as typical for nanostructures generated by spin-coating.

Finally, the gold nanoparticle separation within the printed circles was analyzed by a nearest neighbor distance (NND) algorithm. For samples with highly grained surfaces (NiTi samples and the a-Si samples), the gold nanoparticles arrange themselves in the grooves of the material texture (see Figure 3B1–E1). The NND of these samples, shown in Figure 6, is given for the gold nanoparticles at the boundaries. For better comparison of nanopatterned surfaces with established techniques, the NND for spin-coated poly-Si (poly-Si (ref)) is presented as a reference. Here, a mean separation of 33 nm was determined. The separation distribution is comparably low with a spread of the distribution of about 5 nm. In contrast, the inkjet-printed micelle solution has a much broader distribution and a slightly higher mean NND of 40 nm. Still, the hexagonal order of nanoparticles is present inside the printed circles (Figure 3A1). Interestingly, the mean particle separation distances for a-Si 200 and a-Si 400 sample are in the same range as for poly-Si, but their distribution is much smaller. This is probably a result of the distinct arrangement of the gold nanodots in the texture-induced grooves on these samples.

The distribution for the sputtered NiTi thin film differs from the other samples. The mean NND here is about 27 nm and 50% of the data set for this sample has a small variation of 10 nm. However, the distribution is not symmetric and the mean value is significantly beyond the median of 29 nm compared to the silicon-based samples. This is due to the nanoparticle distribution in the grooves of the texture, where the nanoparticles concentrate. This effect is reminiscent of recently published results, where it was shown that micelles concentrate in the topographically lowest areas on a surface [22].

## Conclusion

In conclusion, we have shown that the combination of BCML with inkjet printing combines the advantages of both methods to form hierarchical micro-nanostructures. The inkjet printing procedure is especially useful on smooth surfaces, such as poly-Si, and defined micropatterns of nanostructures are printed within a few seconds in a very easy-to-handle procedure on large surface areas. In principle, our method can be transferred to any printable substrate. However, for rougher surfaces, the nanostructure inside the printed circles is strongly distorted, and nanostructured patches are formed. Our method can easily be extended to further nanoparticle systems and also to complex printing patterns. Hence, it is relevant in all applications where nanoparticle separation distances and densities need to be controlled at the micrometer scale. For example, the nanoparticles can serve as biomimetic anchorage sites for proteins in biosensor and biomaterial applications.

## Experimental

### Block copolymer micelle nanolithography (BCML)

The samples were functionalized with gold nanoparticles using BCML [13,22,35]. Poly(styrene-*b*-2-vinylpyridine) (PS(79000)-P2VP(36500), 4 mg/mL, Polymer Source, Canada) was dissolved in *o*-xylene (p.A., Merck, Germany) and loaded with hydrogen tetrachloroaurate(III) (Aldrich, Germany) in a molecular ratio of 0.4. The substrates were cleaned in an acetone ultrasonic bath for 15 min and dried. A droplet of 20 μL of the gold-loaded polymer solution was spin-coated onto the poly-silicon substrate as reference at 7000 rpm (WS-650Mz-23NPP, Laurell, USA). The solution was then used for inkjet printing. To remove the micellar polymer, the dry substrates were exposed to a plasma using a mixture of hydrogen and argon gas (10% hydrogen, 90% argon) in a plasma etcher (TePla 100 plasma system, PVA, Germany) at 0.4 mbar and 300 W for 1 h.

### Inkjet printing

A piezoelectric, laboratory scale inkjet printer (Dimatix Materials Printer DMP-2850) was used to generate micropatterns of the micelle solution. Here, the same micelle solution was used as for spin-coating the control sample. The inkjet printer employed a disposable cartridge (DMC-11600), which was made of chemically resistant epoxy, polypropylene, silicone and silicon dioxide. The cartridge was composed of two main components: a jetting and a storage unit. 4 mL micelle solution were filled into the polypropylene bag in the printer storage unit with the help of a syringe. Then, the two units were combined. Regular cleaning cycles were run before, during and after the printing process on a cleaning pad to maintain and improve the printing performance. The cleaning cycle consisted of three actions: blotting, purging and jetting. The substrates were placed on a movable plate with equally spaced holes. This setup provided vacuum to keep the substrate in the desired position. The micellar solution was jetted upon an impulse applied to the jetting module of the cartridge, which is attached to the storage unit. In the jetting module, eight nozzles were embedded in a single row and each nozzle has a channel-type connection to the ink storage unit. To create well-shaped circular droplets on the surfaces, the nozzle frequency and the voltage were optimized iteratively for each substrate between 6–12 kHz and 16–20 V. A pattern of 4 × 4 droplets with a set droplet diameter of 10 µm was printed on the following substrates: poly-silicon, amorphous silicon of 200 nm thickness (prepared by Fraunhofer ISIT, Itzehoe), amorphous silicon of 400 nm thickness (prepared by Fraunhofer ISIT, Itzehoe), 200 nm thick nickel–titanium thin film sputtered on a 4 µm thick copper layer and glass substrate (prepared by Acquandas, Kiel) and free-standing nickel–titanium foil of 50 µm thickness (prepared by Acquandas, Kiel).

### Scanning electron microscopy (SEM) and image analysis

SEM (Supra 55VP, Zeiss, Germany) imaging was carried out at 5 kV using the in-lens detector at a working distance of 5 mm. The SEM images were processed via ImageJ. The nanoparticles were then segmented in the images and indicated as maxima. For analysis of the coordinates of all nanoparticles, the particle analyzer was used (included in ImageJ) and the image was converted to a binary image. Finally, a freely accessible nearest-neighbor detection algorithm was employed for the determination of the nanoparticle distances [36].

### Atomic force microscopy (AFM) imaging and image processing

Atomic force microscopy (AFM) topographic imaging was employed to measure the roughness of the samples. Imaging was performed on a JPK NanoWizard 3 (JPK Instruments AG) operated in ac mode using ACTA cantilevers (spring constant ≈40 N/m, resonance frequency ≈300 kHz; Applied NanoStructuresInc.). Image processing was carried out with JPK SPM Data Processing.
